# Hidradenitis suppurativa in the setting of immune checkpoint inhibitor therapy: A case series

**DOI:** 10.1016/j.jdcr.2025.02.005

**Published:** 2025-03-04

**Authors:** Liam M. Dalton, Sara L. Fossum, Severine Cao

**Affiliations:** Department of Dermatology, University of Michigan Medical School, Ann Arbor, Michigan

**Keywords:** hidradenitis suppurativa, immune checkpoint inhibitor therapy, immunotherapy

## Introduction

Monoclonal antibodies targeting the cytotoxic T-lymphocyte associated protein 4, programmed cell-death protein-1, and its ligands (programmed death ligand-1 and programmed death ligand-2), commonly referred to as immune checkpoint inhibitors (ICI), have become a mainstay in the treatment of a number of different malignancies.^1^ Mechanistically, ICI treatment functions by disabling the co-inhibitory signaling function of the programmed cell death protein-1 protein, its ligands, or cytotoxic T-lymphocyte-associated protein 4 to increase anti-tumor immune responses.^1^ Patients undergoing treatment with these agents can suffer from autoimmune damage to healthy tissues, which can manifest in nearly any organ system in the body. Among immune-related adverse events (irAEs), dermatologic manifestations are common.^2^

Hidradenitis suppurativa (HS) is a common inflammatory dermatologic disorder characterized by the development of abscesses, draining skin tunnels, and scarring affecting intertriginous and anogenital sites.^3^ The pathophysiology of HS is not fully understood, but involves interplay between genetics and the environment with multiple immune pathways playing a role.^3^ ICI treatment has been shown to be associated with exacerbation in a number of autoimmune and inflammatory disorders, including inflammatory bowel disease and psoriasis. However, whether treatment causes or exacerbates HS is not well characterized, and is limited to a small but growing number of case reports.^4^, ^5^, ^6^ Given the central role of inflammation in driving the pathophysiology of HS, its clinical course in the setting of pro-inflammatory ICI treatment warrants further investigation.

In this paper, we describe a case of ICI-induced HS, and then summarize the findings of a retrospective review of patients with HS who received ICI therapy at our institution.

## Index case

The patient was a 78-year-old female with a 10 pack-year smoking history and past medical history of nonalcoholic steatohepatitis complicated by hepatocellular carcinoma. Her hepatocellular carcinoma was initially treated with sorafenib with good response for 5 years; this was discontinued due to gastrointestinal side effects. Progression of HCC disease was noted 1 year later. She underwent stereotactic body radiation therapy but was ultimately started on palliative treatment with bevacizumab and atezolizumab due to disease progression.

**Fig 1:**
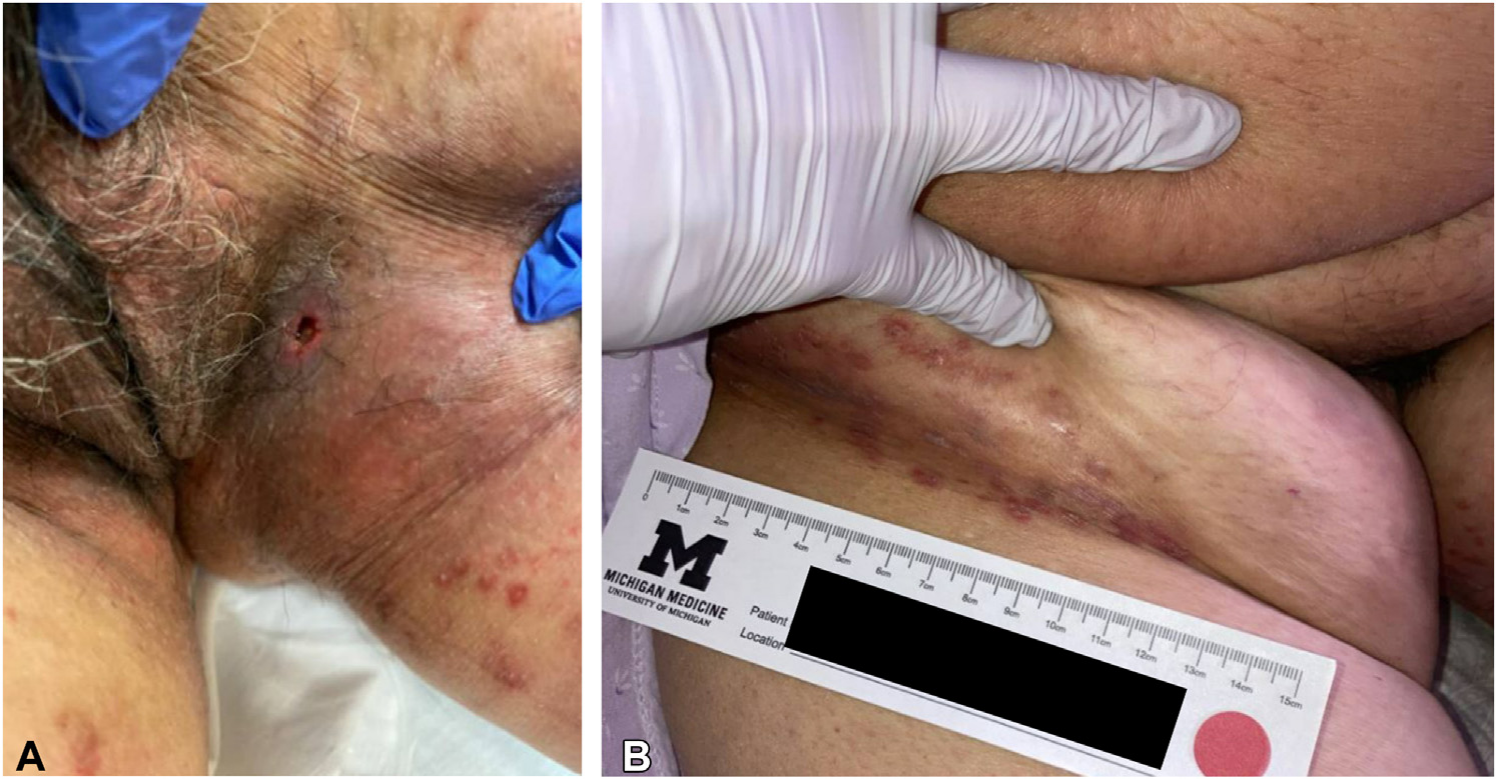
**A,** Eroded inflammatory nodule (photo taken before incision and drainage); (**B**) inflammatory papules and small nodules in the inguinal crease consistent with hidradenitis suppurativa.

Three months after initiation of bevacizumab and atezolizumab, the patient was hospitalized due to abdominal pain and new skin lesions in the bilateral inguinal folds (Fig 1, *A*). The patient denied history of similar lesions or prior HS. Due to suspicion for necrotizing fasciitis or other serious soft tissue infection, general surgery was consulted. While necrotizing fasciitis was excluded, the leading diagnosis was infectious abscess. The patient was treated with incision and drainage and intravenous vancomycin and piperacillin-tazobactam and discharged on oral amoxicillin-clavulanate. Due to concern for ongoing infection, bevacizumab and atezolizumab were discontinued.

Three months after initial presentation, the patient was evaluated in-hospital by the dermatology consult service, who felt the patient’s condition was most consistent with hidradenitis suppurativa due to the finding of skin tunnels, inflammatory papules and nodules, and the locations affected (Fig 1, *B*). Conservative management with benzoyl peroxide 10% wash and topical clindamycin was recommended. The patient transitioned to comfort care only and was discharged to hospice. She passed within a few weeks.

## Case series

We performed a retrospective review of patients at our institution with HS who had received ICI therapy between January 1 2014 and December 31, 2023. We identified patients with International Classification of Disease codes for HS (ICD-9: 705.83, International Classification of Disease codes-10: L73.2) who were on ICI medication in the electronic medical record using DataDirect, a research clinical database at the University of Michigan.^7^ This research project was reviewed and exempted by the University of Michigan Institutional Review Board. The medications screened for were as follows: ipilimumab, tremelimumab, pembrolizumab, nivolumab, atezolizumab, durvalumab, avelumab, cemiplimab, dostarlimab, torpalimab, and retifanlimab. An additional inclusion criterion was a history of having been seen by our institution’s dermatology department, either in an outpatient clinic or by the inpatient consult service. The primary outcome was HS flare, either by patient or physician report, while on ICI treatment, or within 1 year of ICI discontinuation. For patients with prior HS diagnoses, data on Hurley Stage were collected. For patients without a listed Hurley Stage but sufficient documentation, clinic notes were examined, and an estimated Hurley Stage was assigned (denoted by double dagger notation [‡] in Table I). Due to uncertainty in this estimation process, some of these patients were assigned a range of potential Hurley Stages. The clinical courses were also assessed for quiescent versus active disease, with quiescence defined as the absence of a flare within 1 year prior to the initiation of ICI therapy.

**Table I:** Summary of patient characteristics

Case	Sex	Malignancy type	ICI medication	Prior HS diagnosis	Hurley stage	Age at cancer diagnosis	Age at HS symptom onset	Quiescent	Flare	Time on ICI before flare (d)	Prior HS treatments	Flare treatment	Flare leading to ICI discontinuation	irAE
1	F	Ductal carcinoma	Pembrolizumab	No∗	Stage 2	62	15	No	Yes	64	None	Topical antibiotics, oral doxycycline	No	Lichenoid rash
2	F	Hepatocellular carcinoma	Atezolizumab	No†	Stage 1	70	77	N/A	Yes	66	None	Topical antibiotics	Yes	No
3	F	Endometrial adenocarcinoma	Pembrolizumab	Yes	Stage 2-3‡	62	16	Yes	Yes	160	None	Topical antibiotics, oral doxycycline	No	No
4	M	B-cell lymphoma	Pembrolizumab	Yes	Stage 3	30	ND	No	Yes	15	Topical antibiotics, oral antibiotics	Topical antibiotics, oral dexamethasone§	No	No
5	F	Cutaneous T-cell lymphoma	Pembrolizumab	Yes	ID	61	ND	Yes	No	NA	Topical antibiotics	NA	NA	No
6	M	Nonsmall cell lung cancer	Durvalumab	Yes	Stage 2-3‡	53	20s	Yes	No	NA	Oral antibiotics	NA	NA	Morbiliform rash, GI
7	F	Ductal carcinoma	Pembrolizumab	Yes	Stage 1	43	37	Yes	No	NA	Topical antibiotics, oral antibiotics	NA	NA	GI
8	F	Renal cell carcinoma	Nivolumab	Yes	Stage 2-3‡	44	20s	Yes	No	NA	Topical antibiotics, oral antibiotics	NA	NA	No
9	F	Small cell lung cancer	Atezolizumab	Yes	Stage 1	63	11	Yes	No	NA	Topical antibiotics, topical tretinoin, oral antibiotics, intralesional, triamcinolone	NA	NA	No

*HS*, Hidradenitis suppurativa; *ICI*, immune checkpoint inhibitor; *ID*, insufficient documentation; *irAE*, immune-related adverse event; *NA*, not applicable.

∗ No prior diagnosis but endorsed history of symptoms.

† Index case.

‡ Hurley Stage estimated based on clinical documentation.

§ Initiated by oncologist for malignancy treatment.

Using these criteria, a total of 9 eligible patients were identified (Table I). Seven were female and 2 were male. Eight patients (8/9, 88.9%) had a history of HS symptoms or a pre-existing HS diagnosis prior to their cancer diagnosis and ICI treatment. Three patients had Hurley Stage 1 disease, 1 had Stage 2 disease, 1 had Stage 3 disease, and 3 were assessed to have disease between Stages 2-3 based on clinical documentation. Among these 8 patients, 6 (6/8, 75%) had quiescent disease prior to ICI treatment and 2 (2/8, 25%) had active disease.

A total of 4 patients (4/9, 44.4%) experienced an HS flare within the examined timeframe, including 2 who were newly diagnosed with HS; of those, 1 (the index case) had no history of symptoms, whereas the other had a prior history of lesions but no formal diagnosis. All flares occurred within 6 months of starting ICI treatment and occurred while actively undergoing ICI treatment, as opposed to following discontinuation. The median time from initiation of ICI to flare was 65 days. Among the 4 who flared, 2 had pre-existing disease that was considered active. For both, the flare following ICI initiation required an escalation in treatment: Case 3 required the addition of topical antibiotics and oral doxycycline, and Case 4 was treated with oral steroids (which were also being used for their cancer therapy). The HS flare led to ICI discontinuation only in the index case, due to concern for infection. Pembrolizumab was the most commonly prescribed ICI medication (5/9, 55.6%), followed by atezolizumab (2/9, 22.2%), and then nivolumab (1/9, 11.1%) and durvalumab (1/9, 11.1%).

Of the 9 patients, 3 (3/9, 33.3%) experienced an irAE, all 3 of whom subsequently discontinued ICI treatment as a result. Of these patients, 2 (2/9, 22.2%) experienced dermatologic irAEs (Cases 1 and 6), including a patient who separately experienced an HS flare within the timeframe (Case 1). Two patients (2/9, 22.2%) experienced gastrointestinal irAEs. Of the remaining 6 patients, 3 (3/9, 33.3%) discontinued ICI due to progression of malignancy, and 3 (3/9 33.3%) discontinued due to infection, or, in the index case, concern for infection.

## Discussion

ICI treatment is associated with a number of dermatologic manifestations, varying in severity and pathophysiology, including vitiligo, lichenoid dermatitis, psoriasis, bullous pemphigoid, granulomatous diseases, Sweet Syndrome, drug rash with eosinophilia and systemic symptoms, and Stevens–Johnson syndrome.^8^, ^9^, ^10^ Moreover, ICI treatment has been shown to be associated with exacerbation in a number of autoimmune and autoinflammatory disorders.^11^^,^^12^

Here, we present a case of new-onset HS in a 78-year-old patient receiving ICI treatment. Given that the highest incidence of HS occurs from ages 20 to 29 in women, new onset disease at the age of 78 is unusual, and more suggestive that the patient’s disease could have been brought on by the immunologic effects of ICI treatment.^3^^,^^8^ The potential for HS flare following ICI demonstrates the importance of early dermatology involvement in cases of suspected skin and soft tissue infection, especially in medically complex patients. Differentiating inflammatory nodules of HS from a true infection could allow for a “treat-through” approach, with the continuation of ICI treatment in conjunction with symptomatic management by dermatology.^11^

Just under half of identified patients experienced an HS flare while on ICI therapy, with most occurring in those with pre-existing disease. This finding is aligned with prior studies examining the rate of flares in patients with autoimmune or inflammatory disease undergoing ICI therapy: in the case of psoriasis, just over half of patients experienced exacerbation, whereas for patients with autoimmune or inflammatory diseases more broadly, approximately one-quarter of patients experienced a flare.^11^^,^^12^ In evaluating HS flares and their potential relationship to ICI therapy, one challenge is the variable course of HS and the difficulty of identifying whether a flare is due to the ICI or the natural course of the disease. In support of the ICI triggering a flare, we note that all flares occurred within 6 months of treatment initiation. More important, perhaps, is examining how each patient’s disease behaved relative to their baseline. Among the patients with pre-existing HS diagnoses who had active disease, flares on ICI therapy required an escalation of their HS treatment, suggesting a change in their baseline in the context of ICI therapy. This suggests there may be a relationship between HS and ICI therapy, and clinicians prescribing ICI therapy should be cognizant of the potential for flare. Interestingly, whether patients experienced HS flares did not seem to correlate with developing other irAEs.

Fortunately, no flares were severe enough to require ICI discontinuation or rescue therapy and were largely managed without need for further immunosuppression. In our index case, the ICI was discontinued due to concern for an infection. Based on photos at the time of ICI discontinuation (Fig 1), the patient’s HS appeared mild, and we suspect the ICI may not have needed to be stopped if appropriately diagnosed. Similarly, previous studies of autoimmune and inflammatory diseases have found that flares during ICI treatment are typically not severe and are able to be managed using standard treatments without the need for ICI discontinuation.^11^^,^^12^

The large number of pathways involved in HS means there are a variety of ways that the loss of checkpoint inhibition could potentially lead to disease exacerbation.^13^ Prior case reports have speculated on the role of IL-17 activity in the development or exacerbation of HS in the setting of ICI use due to its role in driving HS inflammation as well as potential for downstream interactions with the programmed cell death protein-1 pathway.^5^^,^^6^ Another potential interaction involves the relative lack of certain immunoregulatory myeloid cells, such as CD163^+^ macrophages, in the skin of patients with HS.^13^ These macrophages have been shown to produce programmed death ligand-1 in the tumor microenvironment and are associated with poorer overall survival in patients with certain cancers.^14^^,^^15^ ICI therapy could theoretically lead to local susceptibility to immune activation in patients with a relative deficit of these cells in cutaneous tissues, or potentially sufficient immune activation to cause HS lesions even in patients with normal levels.

Study limitations include the retrospective nature of the study, its small sample size, and lack of standardized documentation of HS flare (especially in setting of inconsistent dermatologic follow-up). Data on treatment escalation occurring in the setting of HS flares may be confounded by these patients undergoing more frequent follow-up and symptom monitoring in the context of oncologic care. As discussed above, it is difficult to say if an HS flare is secondary to ICI treatment, the proinflammatory state induced by the cancer itself, or the natural course of their HS disease. Further study with larger cohorts and prospective data collection is needed to verify and quantify an association between ICI therapy and HS flares. Other future avenues for study include evaluating the association among HS, immunoregulatory pathways, ICI treatment response, and survival.

Overall, our findings suggest that pre-existing HS or concern for new-onset disease should not preclude patients from receiving ICI treatment, but that providers should have a low threshold for seeking dermatologist involvement to optimize both HS and ICI treatment.

## Conflicts of interest

None disclosed.
